# Machine Learning Approaches with Textural Features to Calculate Breast Density on Mammography

**DOI:** 10.3390/curroncol30010064

**Published:** 2023-01-07

**Authors:** Mario Sansone, Roberta Fusco, Francesca Grassi, Gianluca Gatta, Maria Paola Belfiore, Francesca Angelone, Carlo Ricciardi, Alfonso Maria Ponsiglione, Francesco Amato, Roberta Galdiero, Roberta Grassi, Vincenza Granata, Roberto Grassi

**Affiliations:** 1 Department of Electrical Engineering Information Technology, University of Naples Federico II, 80125 Naples, Italy; 2 Medical Oncology Division, Igea SpA, 80013 Napoli, Italy; 3 Department of Precision Medicine, Division of Radiology, University of Campania Luigi Vanvitelli, 80127 Naples, Italy; 4 Italian Society of Medical and Interventional Radiology (SIRM), SIRM Foundation, 20122 Milan, Italy; 5 Division of Radiology, Istituto Nazionale Tumori IRCCS Fondazione Pascale—IRCCS di Napoli, 80131 Naples, Italy

**Keywords:** breast cancer, mammography, breast density, machine learning, radiomics

## Abstract

Background: breast cancer (BC) is the world’s most prevalent cancer in the female population, with 2.3 million new cases diagnosed worldwide in 2020. The great efforts made to set screening campaigns, early detection programs, and increasingly targeted treatments led to significant improvement in patients’ survival. The Full-Field Digital Mammograph (FFDM) is considered the gold standard method for the early diagnosis of BC. From several previous studies, it has emerged that breast density (BD) is a risk factor in the development of BC, affecting the periodicity of screening plans present today at an international level. Objective: in this study, the focus is the development of mammographic image processing techniques that allow the extraction of indicators derived from textural patterns of the mammary parenchyma indicative of BD risk factors. Methods: a total of 168 patients were enrolled in the internal training and test set while a total of 51 patients were enrolled to compose the external validation cohort. Different Machine Learning (ML) techniques have been employed to classify breasts based on the values of the tissue density. Textural features were extracted only from breast parenchyma with which to train classifiers, thanks to the aid of ML algorithms. Results: the accuracy of different tested classifiers varied between 74.15% and 93.55%. The best results were reached by a Support Vector Machine (accuracy of 93.55% and a percentage of true positives and negatives equal to TPP = 94.44% and TNP = 92.31%). The best accuracy was not influenced by the choice of the features selection approach. Considering the external validation cohort, the SVM, as the best classifier with the 7 features selected by a wrapper method, showed an accuracy of 0.95, a sensitivity of 0.96, and a specificity of 0.90. Conclusions: our preliminary results showed that the Radiomics analysis and ML approach allow us to objectively identify BD.

## 1. Introduction

Breast cancer (BC) is the most common cancer among women in the world; about one in eight women will develop breast cancer in their lifetime. Breast cancer survival rates have increased in recent years, and the number of deaths associated with this disease has been steadily declining, largely due to factors such as early detection and personalized treatment approaches. Screening for the early detection of breast cancer is of great interest, as it significantly increases the patient’s chances of survival. It is important to develop techniques to identify key risk factors to benefit from further screening and preventive therapies [1,2,3]. An important role is played by mammography, introduced in the 1960s, which remains the gold standard for breast cancer screening [4]. Over the years, we have witnessed the total replacement of radiographic films with solid-state detectors capable of transforming X-rays into electrical signals to produce the final image directly in a digital format. This technique is called full-field digital mammography (FFDM). Processing and analyzing digital mammograms are significant challenges and can provide quantitative results on breast characteristics. In particular, indices derived from textural patterns of the breast parenchyma can be associated with risk factors. It is established that women with dense breasts have a double risk of developing BC [5,6]. Furthermore, for women with dense breasts, mammography suffers from a loss of sensitivity [7,8]. Breast density (BD) is currently assessed only on a qualitative method by radiologist, therefore based only on a visual assessment, according to the international standard Breast Imaging—Reporting and Data System (BI-RADS) in four classes [9]. However, this qualitative evaluation is subject to intra and inter observer variability. Most of the inter-reader agreement is in the two classifications at the extreme ends of the spectrum (almost entirely fatty and extremely dense), with considerable variation in the agreement between the two middle-density classes.

Therefore, it is desirable to implement a radiomics approach [10], to obtain a quantitative evaluation in order to increase diagnostic accuracy.

The radiomic analysis includes several phases: segmentation of the target region or volume of interest; feature extraction; reduction in the number of characteristics extracted; analysis with the construction of a predictive model and the validation of the results [11,12,13,14,15,16,17,18,19]. The characteristics can be morphological and of a first, second, and higher statistical order.

Such radiomic feature extraction approaches, widely applied in the field of medical imaging [20,21,22,23,24,25,26,27,28,29,30,31,32,33,34,35,36,37,38,39,40,41,42,43,44,45,46,47,48,49,50,51,52,53,54,55,56,57,58,59,60], can be combined with the use of regression and classification techniques, with particular regard to machine learning (ML) algorithms, which have been widely used in the medical and biomedical fields for various purposes, ranging from the study of bio-signals and bioimaging [61,62,63,64,65,66,67,68,69] to the analysis of health processes [70,71,72,73].

Today, the main scenario is the oncological environment, since the characteristics of radiomics provide data on the tumor microenvironment that could be related to the risk of developing cancer, histological grade, prognosis, response to treatment and survival in countless tumors [20,21,22,23,24,25,26,27,28,29,30,31,32,33,34,35,36,37,38,39,40,41,42,43,44,45,46,47,48,49,50,51,52,53,54,55,56,57,58,59,60].

Given the importance of breast density as a risk factor for BC and increasing attention to screening strategies based on density and considering the low accuracy that could be correlated with a qualitative assessment, due to the inter- and intra-reader variability in radiologists’ interpretation, the aim of this study was to combine radiomics and ML in order to explore the possibility of correlating statistical and textural characteristics of digital images (such as interactions between pixels or gray levels) with BD [74,75,76,77], to obtain a more objective evaluation. The accuracy of the classifiers on ML algorithms was then evaluated to estimate the breast density class.

## 2. Methods

### 2.1. Study Population

This observational retrospective study was approved by the ethics committee of the University “Luigi Vanvitelli”, Naples, Italy, with deliberation n. 469 of 23/07/2019 and, informed consent was waived by the ethics committee. All methods were carried out according to National regulations and guidelines.

We enrolled all consecutive women, who underwent mammography for breast cancer screening programs, at the Breast Unit of the University Hospital “Luigi Vanvitelli”, Naples, from June 2020 to November 2020.

A validation cohort, consisting of a total of 51 patients, obtained from the Breast Team of “Villa Fiorita”, Capua, Italy, was considered in this study and enrolled.

Moreover, we assessed breast structures according to BI-RADS 5th [7].

### 2.2. Digital Mammograms Acquisition

All the women from the internal cohort underwent two-view bilateral full-field digital mammography according to the standard protocol, using Giotto Class (IMS GIOTTO S.p.A., Sasso Marconi, Bologna, Italy). All the women from the external cohort underwent two-view bilateral full-field digital mammography according to the standard protocol, using GE SENOGRAPHE ESSENTIAL (General Electric Company, Milano, Italy).

For both the internal and external cohort, patient images were acquired both in cranio-caudal (CC) and medio-lateral-oblique (MLO) views, on the right and left sides.

The processing is carried out on the “FOR PRESENTATION” images, i.e., raw images, because being proportional to the attenuation of the X-rays on the breast tissue, they retain the original information on the attenuation of the X-rays and therefore are more suitable for quantitative analyzes.

In total, four images are obtained for each patient. The images have a resolution of 2729 × 3580 (on average for all images) and a laptop with a 2.40 GHz Intel Core i5-9300H CPU, 8 GB of RAM, and the NVIDIA GeForce GTX 1650 graphics card was used for the image processing, training, and machine testing Learning algorithms.

### 2.3. Image Pre-Processing

Raw images undergo two main stages of preprocessing: image enhancement, to improve the quality and information content of the original data, and breast segmentation, to identify the air-tissue interface and remove the pectoral muscle, mainly in the MLO configuration.

In the first phase, the equalization of the histogram, which consists in applying a logarithmic transformation in order to enhance the pixels of the image and display the different structures of which the breast is composed, and a gray scale normalization with a is used as a range of values between [0,1]. After seeing the image of the breast, three areas can be distinguished: pectoral muscle, which appears whiter than the rest of the breast; mammary tissues, consisting mainly of adipose and fibroglandular tissue; and the region occupied by the air surrounding the breast, also called the bottom.

The purpose of the second step is to isolate only the breast tissue, i.e., the Region of Interest (ROI), eliminating both the pectoral muscle and the background.

Segmentation was achieved using a software tool implemented in MATLAB R2007a (The MathWorks, Inc., Natik, MA, USA), manually drawn by two expert radiologists (22 and 15 years of breast imaging experience, respectively); the segmentation was performed by the two radiologists first separately and then together and in accordance with each other.

### 2.4. Features Extraction and Features Selection

A total of 229 features were extracted: 1–112 features of Haralick; 113–157 features of Law; 158–185 features of Run Length; 186–215 features of Wavelet; 216–227 features of the histogram; 228 Fractal dimensions; 229 Local binary patterns. The textural features were calculated according to the Image Biomarker Standardization Initiative [78].

To eliminate redundant features, both a selection of filtering features based on correlation and a selection of wrapper features were used in this study. Filter feature selection is a method of applying statistical measures by assigning a certain score to each feature. The characteristics are ranked by score and selected or removed from the dataset according to the classification. Therefore, a filter characteristics selection method that searches for correlation between characteristics using functions *cor* and find *Correlation* of the *caret* package [79] with a cutoff set to 0.9 [80] was a performed.

The wrapper’s feature selection method is to set up the problem of selecting a suitable set of features as a search problem between sets of different combinations of features. To select the best set, you can use a predictive model in which you assign a score to each combination. The research process can be carried out following different approaches, some of these are for example methodical algorithms, such as “best-first” or stochastic methods such as “random-hill climbing” or heuristic methods. An example of a wrapper method is Recursive Feature Elimination (RFE).

The implementation of the wrapper features selection was achieved using the *rfe* and *rfeControl* functions of the *caret* package [79] by choosing an algorithm as a model to evaluate the accuracy of a Random Forest (RF) and choosing equal to 100 the number of features to be selected among the starting point. This value was chosen empirically, evaluating that the maximum accuracy in relation to the number of features chosen is already reached before the selected value, making it unnecessary to select multiple features.

### 2.5. Statistical and Machine Learning Analysis

A nonparametric Mann Whitney test was performed to identify statistically significant differences in radiomic metrics among groups for age and BMI.

A *p*-value < 0.05 was considered significant.

The data obtained through the extraction of features and the application of a feature selection algorithm are classified through different ML algorithms (support vector machine (SVM), linear discrimination analysis (LDA), artificial neural network (ANN), decision tree (DT), Random Forest Tree) [81].

To solve the problem of the unbalanced dataset, we transformed it into a binary classification problem by associating patients with classes A and B and patients with classes C and D [77]. The division into training and testing on the dataset was made by partitioning the data and leaving 80% for training and 20% for model testing.

The performance of a classification can be summarized using the Confusion Matrix, while for binary classification, it is also possible to add the ROC curves (Receiver operation features).

Sensitivity, specificity, and accuracy were calculated. Sensitivity is the portion of correctly identified true positives. Specificity is the portion of true negatives correctly identified. Accuracy is the parameter that summarizes the effectiveness of the classification by evaluating the proportion of correct predictions (both positive and negative) on the total number of cases examined.

The classification analysis was cross-validated using the 10-fold cross-validation approach, and median values of AUC, accuracy, sensitivity, and specificity were obtained.

The best model was chosen considering the highest area under the ROC curve and the highest accuracy. The McNemar test was used to assess the statistical significance of dichotomous tables. A *p*-value < 0.05 was considered significant.

All analyses were performed with the R-Studio Version 1.3.959 (https://www.rstudio.com/ Accessed date 14 March 2022) [82].

## 3. Results

A total of 168 patients from the internal cohort aged 38 to 84 were enrolled over a 5-month period from June 2020 to November 2020.

A total of 51 patients from the validation cohort aged 37 to 80 were enrolled over a 4-month period from January 2021 to April 2021. Patients’ characteristics are reported in Table 1 and Table 2.

To reduce the computational problem in the classification analysis considering the small size for the four classes, we transformed the dataset into a binary dataset.

According to the binary classification, the internal dataset is transformed into 99 patients with densities A and B: fat breast; and 69 patients with densities C and D: dense breast.

While the external validation cohort included 27 fat breast and 24 dense breast.

The results related to the features selection correlation method was a subclass of 35 textural characteristics, as shown in Table 3. No statistically significant difference (*p*-value > 0.05 at Kruskal Wallis test) was found in the median values of these 35 selected textural features for age subgroups (15–44; 45–54; 55–64; 65–78; ≥78 years). Moreover, no statistically significant difference (*p*-value > 0.05 at Kruskal Wallis test) was found in median values of these 35 selected textural features for BMI subgroups (Normal (<25 kg/m^2^); Overweight (25–29 kg/m^2^); Obese (≥30 kg/m^2^)).

Following the application of the Wrapper feature selection, a subclass of 7 features was obtained and reported in Table 4. No statistically significant difference (*p* value > 0.05 at Kruskal Wallis test) was found in the median values of these 7 selected textural features for age subgroups (15–44; 45–54; 55–64; 65–78; ≥78 years). Moreover, no statistically significant difference (*p* value > 0.05 at Kruskal Wallis test) was found in the median values of these 7 selected textural features for BMI subgroups (Normal (<25 kg/m^2^); Overweight (25–29 kg/m^2^); Obese I (≥30 kg/m^2^)).

Only the Energy Haralick D30 structural feature was identified as a significant predictor by the two different feature selection methods.

**Figure 1:**
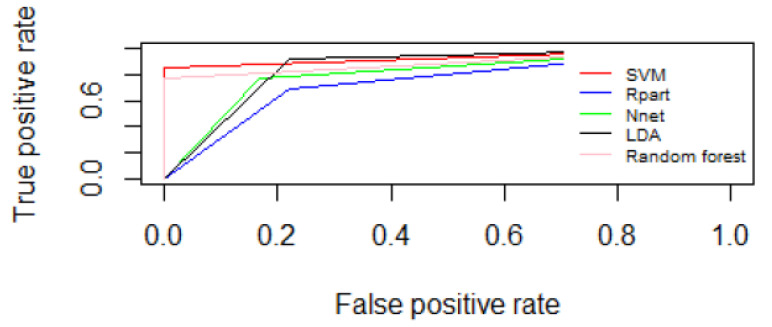
ROC curves between fat and dense breasts considering the 35 selected textural features by means of filter feature selection correlation method.

**Figure 2:**
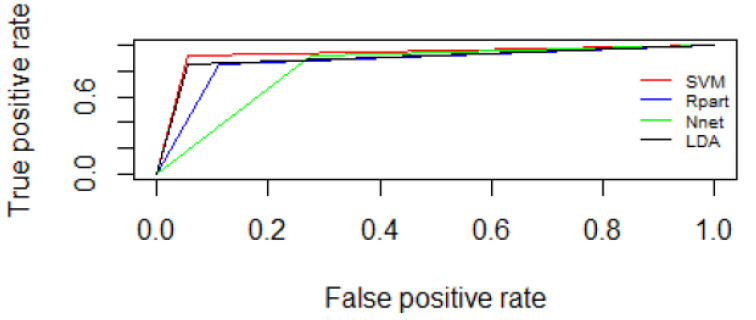
ROC curves between fat and dense breasts considering the 7 features selected with the wrapper features selection method.

Table 5 and Table 6 reported the preliminary results of the ML algorithms tested using the two feature selection approaches: the filter feature selection correlation method and the feature selection wrapper method. Figure 1 and Figure 2 report ROC curves between fat and dense breasts considering the 35 selected textural features by means of filter feature selection correlation method and ROC curves between fat and dense breasts considering the 7 features selected with the wrapper features selection method.

The accuracy varied between 74.15% and 93.55% using the filter feature selection correlation method; while using a feature selection wrapper method, the range of accuracy was 87.10% and 93.55%. In both cases, the SVM algorithm, with k = 0.8675, reached the best results (accuracy of 93.55% and a percentage of true positives and negatives equal to TPP = 94.44% and TNP = 92.31%). The F1 score for the SVM algorithm was equal to 0.86. The training and prediction time was a few seconds.

Considering the external validation cohort, the SVM, including the 35 predictors obtained from the feature selection correlation method, showed an accuracy of 0.94, a sensitivity of 0.97, and a specificity of 0.88.

Considering the external validation cohort, the SVM, as the best classifier with the 7 features selected by the wrapper method, showed an accuracy of 0.95, a sensitivity of 0.96, and a specificity of 0.90. The best SVM classifier had the following configuration setting: Linear SVM; kernel function: linear; kernel scale: automatic; box constraint level: 1; multiclass method: one-vs-one; standardize data: true; optimizer options; and hyperparameter options disabled.

## 4. Discussion

Breast density is reflected as an independent increased risk factor for breast cancer, and denser breasts can mask the presence of cancer, especially in the early stages, when it is critically important to make the diagnosis to ensure a positive prognosis for the patient. The purpose of the study is to define a quantitative analysis of mammographic images to support the qualitative assessment of breast density by radiologists. Although evaluation of BD has shown several values for estimating BC risk, a qualitative or BI-RADS approach is highly subjective to inter- and intra-observer variability [83,84,85]. In contrast, radiomics evaluation of mammographic data could allow a more complex characterization of the intricacy and morphologic organization of the parenchymal patterns.

Radiomics analysis is based on the conversion of medical images to higher dimensional data by using computer-based algorithms. Several authors showed that Radiomics data, when correlated with clinical-pathological outcomes, could allow a noninvasive and cost-effective approach to favor precision medicine [86,87,88,89,90,91,92,93,94,95,96]. Preliminary studies showed that radiomics features were correlated with BC independent of BD and, therefore, had the potential to augment BD in assessing a woman’s risk of developing cancer [97,98,99].

The possibility of objectively identifying risk factors, such as BD, allows for better risk stratification of the patient and better management. We assessed 168 patients, 99 patients with fat breast and 69 patients with dense breast. A total of 229 features were extracted, and after a selection correlation method, we obtained a subclass of 35 textural characteristics. Thanks to the extraction of the texture characteristics from the image dataset, it was possible to evaluate a binary classification using ML algorithms, which led to results that show a good agreement with the radiologist’s report; a maximum accuracy of 93.55% was obtained in the internal cohort with an SVM method and percentage of true positives and negatives equal to TPP = 94.44% and TNP = 92.31%. Considering the external validation cohort, the SVM, as the best classifier with the 7 features selected by the wrapper method, showed an accuracy of 0.95, a sensitivity of 0.96, and a specificity of 0.90.

In line with our results, also Kantos et al. [99] assessed phenotypes of mammographic parenchymal complexity by using radiomic features and evaluated their associations with breast density and other breast cancer risk factors. They showed that an unsupervised clustering identified four phenotypes with increasing parenchymal complexity that were reproducible between training and test sets. Breast density was not strongly correlated with the phenotype category. The low- to intermediate-complexity phenotype had the lowest proportion of dense breasts, whereas similar proportions were observed across other phenotypes. In the independent case-control sample, phenotypes showed a significant association with BC, resulting in a higher discriminatory capacity when added to a model with BD and body mass index.

Li et al. [100] combined the characteristics of normal parenchyma from the contralateral breast with radiomic features of breast tumors to improve the accuracy in the diagnosis of BC. They showed that the performance of the combined lesion and parenchyma classifier was better than that of the lesion features alone. Overall, six radiomic features, such as spiculation, margin sharpness, size, circularity from the tumor feature set, and skewness and power law beta from the parenchymal feature set, were selected [100].

Being able to objectively assess the risk of BC would be an important step to reducing the effects of this disease, along with its mortality rate. Yala et al. [101] tried to develop a model that used mammograms as risk factors (BD and patient age) to predict the risk of BC. They used 71,689 images for training (2732 positive vs. 68,957 negative), 8554 for validation (316 positive vs. 8238 negative), and 8869 for testing (269 positive vs. 8282). The authors proposed 3 models. The first is based on a logistic regression, containing only the risk factors; the second is based only on images, using a ResNet18 architecture. The last, a hybrid DL model that combines data from the previous two models. The hybrid model achieved an AUC of 0.70 [101].

Our results may be considered preliminary, on a still limited dataset, but they highlight the possibility of obtaining promising results with a larger dataset.

A future endpoint could be to correlate imaging metrics with clinical features, such as the age of a patient that could influence breast tissue and the possibility to obtain a hybrid model to predict the risk of BC.

The present study has several limitations. It was performed in a retrospective manner at a single academic institution, and the results may not generalize to other practice settings. We did not assess the interreader agreement for the BD assessment. In addition, we did not evaluate the BC rate considering BD categories. Future studies should be aimed at evaluating better BC detection in the context of dense breasts.

## 5. Conclusions

Our results showed that the Radiomics analysis and ML approach can objectively identify BD. Thanks to the extraction of the texture characteristics from the image dataset and a binary classification using ML algorithms we obtained an accuracy of 93.55% with an SVM method and a percentage of true positives and negatives equal to TPP = 94.44% and TNP = 92.31%.

## Figures and Tables

**Table 1:** Number of patients across breast density and age group.

Age Group (yrs)	Breast Density			
	A	B	C	D
Internal Cohort				
15–44	0	6	2	7
45–54	4	18	25	9
55–64	8	41	5	15
65–78	5	14	0	5
>78	0	3	1	0
Sum	17	82	33	36
Validation Cohort				
15–44	0	1	1	2
45–54	1	2	2	1
55–64	1	4	3	3
65–78	2	8	5	2
>78	2	6	3	2
Sum	6	21	14	10

**Table 2:** Number of patients across breast density and BMI.

Age Group (yrs)	Breast Density			
	A	B	C	D
Internal Cohort				
Normal (<25 kg/m^2^)	12	15	9	8
Overweight (25–29 kg/m^2^)	9	17	16	10
Obese (≥30 kg/m^2^)	11	35	13	13
Sum	32	67	38	31
Validation Cohort				
Normal (<25 kg/m^2^)	4	2	3	4
Overweight (25–29 kg/m^2^)	6	3	4	5
Obese I (≥30 kg/m^2^)	6	6	3	5
Sum	16	11	10	14

**Table 3:** 35 features selected with the filter features selection method.

Variable Number	Variable Name
V687	Fractal dimensions
V685	95th percentile Hist feat
V684	mean 5th percentile Hist feat
V682	Entropy Hist feat
V679	Std Hist feat
V678	Min histogram value
V677	Max histogram value
V674	Kurtosis Diagonal comp. Wavelet 5° iteration
V672	Kurtosis Vertical comp. Wavelet 5° iteration
V671	Variance Vertical comp. Wavelet 5° iteration
V670	Kurtosis Horizontal comp. Wavelet 5° iteration
V669	Variance Horizontal comp. Wavelet 5° iteration
V669	Kurtosis Diagonal comp. Wavelet 4° iteration
V668	Kurtosis Vertical comp. Wavelet 4° iteration
V666	Variance Vertical comp. Wavelet 4° iteration
V665	Kurtosis Horizontal comp. Wavelet 4° iteration
V664	Kurtosis Diagonal comp. Wavelet 3° iteration
V662	Kurtosis Vertical comp. Wavelet 3° iteration
V660	Kurtosis Horizontal comp. Wavelet 3° iteration
V656	Kurtosis Diagonal comp. Wavelet 2° iteration
V654	Kurtosis Vertical comp. Wavelet 2° iteration
V652	Kurtosis Horizontal comp. Wavelet 2° iteration
V646	Kurtosis Horizontal comp. Wavelet 1° iteration
V641	Run Percentage RL 180 degrees
V636	Low Gray Level Run Emphasys RL 90 degrees
V633	Run Percentage RL 90 degrees
V631	Short Run Emphasys RL 90 degrees
V618	Short Run Emphasys RL 0 degrees
V607	Mean Map8 Law
V598	Standard Deviation Map6 Law
V569	Haralick D120 Measure of correlation I
V530	Haralick D30 Energy
V518	Haralick D15 Correlation
V473	Maximal Correl. Coeff. (Mean 0°, 45°, 90°, 135°)
V469	Haralick Entropy (Mean 0°, 45°, 90°, 135°)

**Table 4:** 7 features selected with the wrapper features selection method.

Variable Number	Variable Name
V558	Energy Haralick D120
V552	Entropy Haralick D60
V544	Energy Haralick D60
V566	Sum Entropy Haralick D120
V530	Energy Haralick D30
V614	Skewness Map9 Law
V516	Energy Haralick D15

**Table 5:** Classification evaluation metrics between fat and dense breasts with elimination of correlate features (filter feature selection correlation method).

Method	Accuracy	95% CI	Kappa	Sensitivity	Specificity	*p* Value
SVM	0.93	0.79–0.99	0.86	1.00	0.85	<0.001
Random Forest Tree	0.91	0.79–0.99	0.86	1.00	0.85	<0.001
LDA	0.84	0.66–0.94	0.68	0.778	0.92	<0.01
ANN using the nnet package	0.74	0.55–0.88	0.47	0.778	0.69	<0.01
Decision Tree using the Rpart package	0.74	0.55–0.88	0.47	0.778	0.69	<0.01

Note. SVM: support vector machine, LDA: linear discrimination analysis, ANN: artificial neural network.

**Table 6:** Classification evaluation metrics between fat and dense breasts with RFE.

Method	Accuracy	95% CI	Kappa	Sensitivity	Specificity	*p* Value
SVM	0.93	0.79–0.99	0.87	0.94	0.92	<0.001
LDA	0.90	0.74–0.98	0.80	0.94	0.85	<0.01
ANN using the nnet package	0.68	0.49–0.83	0.34	0.83	0.46	<0.01
Decision Tree using the Rpart package	0.87	0.70–0.96	0.73	0.89	0.85	<0.01

Note. SVM: support vector machine, LDA: linear discrimination analysis, ANN: artificial neural network.

## Data Availability

The data presented in this study is available in this article.
